# A Scoping Review of Adult Inpatient Satisfaction with Mental Health Services

**DOI:** 10.3390/healthcare11243130

**Published:** 2023-12-09

**Authors:** Hossam Elgendy, Reham Shalaby, Ernest Owusu, Nnamdi Nkire, Vincent I. O. Agyapong, Yifeng Wei

**Affiliations:** 1 Department of Psychiatry, Faculty of Medicine and Dentistry, University of Alberta, Edmonton, AB T6G 2R3, Canada; rshalaby@ualberta.ca (R.S.); eowusu2@ualberta.ca (E.O.); nnamdi.nkire@albertahealthservices.ca (N.N.); agyapong@ualberta.ca (V.I.O.A.); yifeng.wei@ualberta.ca (Y.W.); 2 Addiction and Mental Health, Alberta Health Services, Edmonton, AB T5J E34, Canada; 3 Department of Psychiatry, Faculty of Medicine, Dalhousie University, Halifax, NS B3H 4R2, Canada

**Keywords:** satisfaction, appreciation, contentment, experience, inpatient, mental health, hospital care

## Abstract

Patient satisfaction with hospital services has been increasingly discussed as an important indicator of healthcare quality. It has been demonstrated that improving patient satisfaction is associated with better compliance with treatment plans and a decrease in patient complaints regarding doctors’ and nurses’ misconduct. This scoping review’s objective is to investigate the pertinent literature on the experiences and satisfaction of patients with mental disorders receiving inpatient psychiatric care. Our goals are to highlight important ideas and explore the data that might serve as a guide to enhance the standard of treatment and patient satisfaction in acute mental health environments. This study is a scoping review that was designed in adherence with the Preferred Reporting Items for Systematic Reviews and Meta-Analyses Extension for Scoping Reviews (PRISMA-ScR) statement. A systematic search was conducted in the following databases: PubMed, MEDLINE, PsycINFO, CINAHL, and EMBASE. A comprehensive review was completed, including articles from January 2012 to June 2022. Qualitative and quantitative studies were included in this review based on our eligibility criteria, such as patient satisfaction as a primary outcome, adult psychiatric inpatients, and non-review studies published in the English language. Studies were considered ineligible if they included nonpsychiatric patients or patients with neurocognitive disorders, review studies, or study measure outcomes other than inpatient satisfaction. For the eligible studies, data extraction was conducted, information was summarized, and the findings were reported. A total of 31 studies representing almost all the world’s continents were eligible for inclusion in this scoping review. Different assessment tools and instruments were used in the included studies to measure the level of patients’ satisfaction. The majority of the studies either utilized a pre-existing or newly created inpatient satisfaction questionnaire that appeared to be reliable and of acceptable quality. This review has identified a variety of possible factors that affect patients’ satisfaction and can be used as a guide for service improvement. More than half of the included studies revealed that the following factors were strongly recommended to enhance inpatient satisfaction with care: a clear discharge plan, less coercive treatment during the hospital stay, more individualized, higher quality information and teaching about the mental disorder to patients by staff, better therapeutic relationships with staff, and specific treatment components that patients enjoy, such as physical exercise sessions and music therapy. Patients also value staff who spend more time with them. The scope of patient satisfaction with inpatient mental health services is a growing source of concern. Patient satisfaction is associated with better adherence to treatment regimens and fewer complaints against health care professionals. This scoping review has identified several patient satisfaction research gaps as well as important determinants of satisfaction and how to measure and utilize patient satisfaction as a guide for service quality improvement. It would be useful for future research and reviews to consider broadening their scope to include the satisfaction of psychiatric patients with innovative services, like peer support groups and other technologically based interventions like text for support. Future research also could benefit from utilizing additional technological tools, such as electronic questionnaires.

## 1. Introduction

Patient satisfaction began to receive scientific attention in the 1950s when it was realized that higher patient satisfaction was associated with better patient adherence to doctors’ prescriptions and medication consumption. It was also found to be associated with a decrease in patient complaints regarding professional misconduct [1]. The majority of the funds allocated for mental health services are often directed to staffing and beds in psychiatric hospitals in many different parts of the world; however, there is a significant disregard for the evaluation of both out- and inpatient mental health services as rated directly by patients [2]. Although focusing on the treatment gap, such as the lack of or insufficient quantity of services, is important in the mental health agenda, the quality of care from the perspective of people using it is crucial as well [2]. High satisfaction and high-quality care are frequently correlated. Positive experiences help patients achieve better results in terms of their mental health. People are more likely to actively participate in their treatment plans when they are happy with the care they receive, which enhances their general well-being. Furthermore, the stigma attached to obtaining and using mental health services can be lessened with positive experiences in mental health facilities. Positive interactions increase the likelihood that people will share their experiences, which can foster a more understanding and encouraging community [3]. Additionally, in any healthcare relationship, trust is essential. Patients’ satisfaction with inpatient mental health services contributes to increased trust in medical professionals and the health system. This trust is essential for ongoing collaboration between patients and healthcare providers [3,4,5]. In this context, the increased marketing of healthcare services has prompted the creation of tools used for evaluating patient satisfaction as an indicator of healthcare quality and the efficiency of the healthcare system [6,7]. Although there is no agreed-upon definition of “patient satisfaction”, it is occasionally used as a subjective measure of whether a patient’s expectations for medical contact were met [8]. In other sources, it is described as a measurement of the degree to which a patient is happy with the care they receive from their doctor, a healthcare facility, or another healthcare professional [7]. The lack of a universally agreed-upon definition of patient satisfaction can be attributed to several factors such as subjectivity and cultural difference: which means patient satisfaction varies from person to person and is inherently subjective. It can be difficult to come up with a single, all-encompassing term that encompasses everyone’s viewpoints because different people may value different aspects of their healthcare experiences more than others. Also, the complexity of the healthcare process, where patients, healthcare providers, administrators, insurers, and other stakeholders are all involved in this complex and multifaceted system and priorities and expectations of each of these stakeholders could differ, could make it more difficult to come up with a standard definition. Additionally, healthcare evolution is very dynamic and ever-changing; hence, patient expectations and experiences may be impacted by new technologies, treatment options, and modifications to healthcare delivery models that may influence patient satisfaction as healthcare changes [9,10]. The relevance of reporting patient satisfaction as a component of studies assessing treatment outcomes has recently increased. There is no doubt that this crucial aspect of patient care must be accurately assessed, but the techniques and metrics required to do so have not yet been sufficiently established, and up to now, no conclusion could be drawn regarding factors leading to higher patient satisfaction. Several factors contribute to inpatient satisfaction, including the following: quality of care, where patients often evaluate the efficacy of medical interventions and the skill of healthcare professionals; communication is another factor, where clear and effective communication between healthcare providers and patients is crucial; accessibility, which is a patient’s level of satisfaction that is affected by how simple it is for them to obtain healthcare services, including, waiting periods, and the location of the facility; empathy and compassion, where patients value medical professionals who exhibit these qualities because they indicate that they are aware of and concerned about their well-being; dignity and respect: upholding patients’ dignity, honoring their cultural and personal preferences, and treating them with respect all contribute to their general satisfaction; and finally, facility and environment: the hospital physical layout, level of comfort, cleanliness, and general ambience can all affect how satisfied patients are [7,11,12,13]. There is no clear instruction on how to utilize any of the several satisfaction scales and metrics available to psychiatric inpatients for the assessment of inpatient satisfaction [8,9,10]. It is difficult to provide a general overview of patients’ satisfaction with inpatient care because different instruments and methodological approaches were used in different studies to measure satisfaction [6]. There are various methods and tools for measuring patient satisfaction, including anonymous survey questionnaires, feedback forms, and interviews. Different healthcare organizations may use different instruments, making it challenging to standardize the measurement process and arrive at a universally accepted definition or instrument [14].

The primary aim of the studies included in this scoping review was the assessment of inpatient satisfaction against various factors in the healthcare facility, which are specific to each individual study or a group of studies.

To this end, the aim of this scoping review is to explore the published literature in the last ten years that is relevant to the experience and satisfaction of patients with mental health disorders in inpatient psychiatric units in various regions of the world and to shed light on the different tools of assessments that are used for this purpose, in addition to identifying the global distribution of the published studies in this field. We hope to provide information, identify the gaps that need to be filled, and explore the key concepts that may help to improve the quality of care and satisfaction in acute psychiatric settings.

## 2. Methods

This scoping review was structured in adherence with the Preferred Reporting Items for Systematic Reviews and Meta-Analyses Extension for Scoping Reviews (PRISMA-ScR) statement [15]. The review followed Arksey and O’Malley’s five-stage approach to scoping reviews [16]. A publication search was conducted in five databases, including PubMed, MEDLINE, CINAHL PsycINFO, and EMBASE. A thorough analysis that included publications from January 2012 to June 2022 was carried out. The rationale behind choosing this particular time frame was to ensure that the articles were recent and that a sufficient number of pertinent studies were available. Extracts from pertinent papers were evaluated and analyzed. Finding a set of publications that concentrated on inpatient satisfaction with mental health care was the main objective of the article screening process. This review covered both qualitative and quantitative research. Review studies such as (systematic reviews, meta-analyses, or scoping reviews), Psychometric research, theses or protocols, publications written in a language other than English, and articles with a theoretical or opinion focus were all excluded from this review. Peer-reviewed journals published each of the featured publications.

The publications were assessed with the help of three reviewers/authors (HE, RS, EO), who independently screened the titles and abstracts and examined all full-text articles that met the inclusion criteria. Each piece required independent assessment by two reviewers, and conflicts were resolved by thorough discussions during personal or virtual meetings; a third reviewer joined to solve the conflicts if consensus was not made between the two reviewers.

### 2.1. Inclusion and Exclusion Criteria

**Studies were considered eligible when the following criteria were met:**

(1) Outcome: Measurement/studying psychiatric inpatients’ satisfaction as a primary outcome;
(2) Setting: Inpatient psychiatric care (to explore gaps and help in improvement);
(3) Place: All countries and regions were included (for broadening the study scope);
(4) Population:
  - Adult patients (more than or equal to 18y) (we chose to focus on adult age group);
  - Any mental health diagnosis of adult population, including addiction and alcohol use disorder, for purpose of broadening the study scope;
(5) Type of study:
  - Articles published in English language;
  - Studies published within the last 10 years (to ensure the recency of evidence);
  - Quantitative or qualitative (for better understanding of patients’ viewpoints);
  - Individual (non-review) studies, such as cross-sectional, randomized controlled trials (RCTs), cohort studies, case–control studies;
(6) Type of the assessed service:
  - Structural design (e.g., hospital design and beds);
  - Hospital environment (e.g., cleanliness and food);
  - Therapeutic lines (e.g., clozapine, electro-convulsive therapy (ECT), exercise);
  - Personnel (e.g., nurses and doctors).

**Studies were considered ineligible when any of the following criteria were met:**

(1) Setting: Non-psychiatric inpatient units, such as
  - Outpatient mental health services;
  - Emergency departments;
  - Day hospital;
  - Long-term care;
  - Rehabilitation care;
(2) Outcome:
  - Studies measure outcomes other than inpatient satisfaction;
  - Satisfaction as a secondary outcome;
  - Studies measure the feasibility or acceptability of satisfaction assessment programs;
(3) Population:
  - Non-psychiatric patients;
  - Non-patients, e.g., relatives, caregivers, healthcare workers, children, and adolescents;
  - Patients diagnosed with neurocognitive disorders (dementia and delirium) or primary pediatric mental health conditions (e.g., Tourette syndrome);
(4) Type of study:
  - Studies published before 2012;
  - Review studies, e.g., systematic reviews, meta-analyses, scoping reviews, psychometric studies, theses, or protocols;
  - Studies in a language other than English;

### 2.2. Search Terms

The search strategy embraced a combination of keywords, including mental health, psychiatric inpatients, and hospital care, and descriptors, including satisfaction, appreciation, contentment, gratification, and experience. These search terms were combined using the AND Boolean operator, with each individual term connected with the OR Boolean operator within each search term.

### 2.3. Data Extraction

For eligible studies, the following data were extracted using a data extraction form: author name and year of publication, type of study, country of study, diagnosis, number of participants, aim of the study, tools for satisfaction assessment, and study results.

## 3. Results

**Figure 1:**
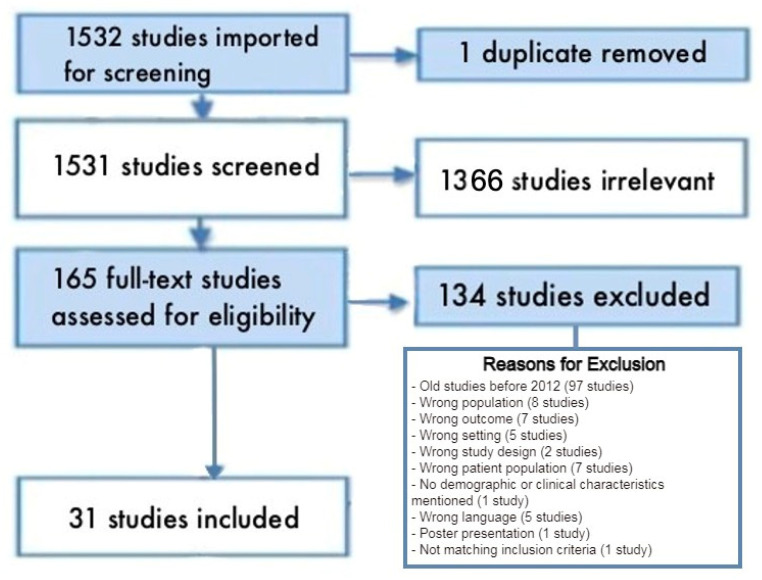
PRISMA flow diagram.

The search strategy identified a total of 1532 studies from the electronic databases searched using Covidence software 2022 (Covidence.org: Melbourne, VIC, Australia). Covidence is a web-based software platform designed to assist in the systematic review process in academic research. Systematic reviews involve the comprehensive and structured analysis of a specific research question by gathering and evaluating relevant studies. Covidence aims to streamline and facilitate this process for researchers [17]. One article was automatically reviewed by Covidence software and eliminated for duplication. Based only on the title and abstract of the remaining 1531 papers, 165 pieces of research that met the authors’ (HE, EO, RS) eligibility requirements were found. After full-text screening phase, 134 studies were excluded, leaving a total of 31 studies that were eligible to be included in this scoping review. The information is summarized in the PRISMA flow diagram (Figure 1).

**Figure 2:**
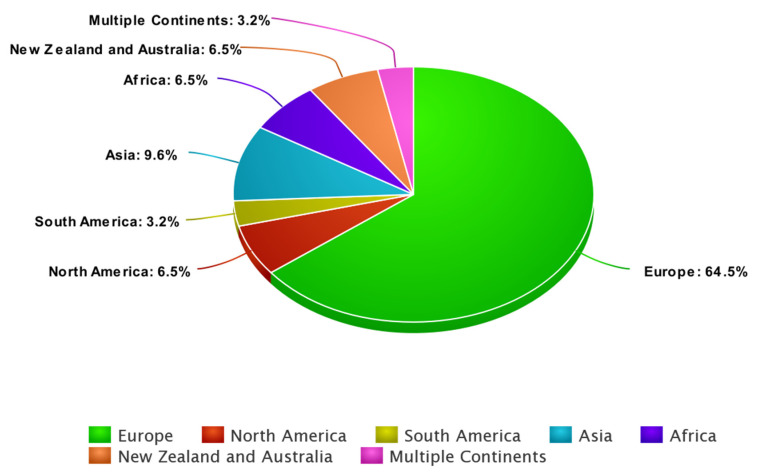
Summary of continents selected for the review.

**Continent distribution of the studies**: Figure 2 demonstrates the summary of the global distribution of the studies included in this review, according to the place of the study (continent). From the figure, most of the studies were conducted in Europe (*n* = 20, 64.5%), while the other continents were represented to a lesser extent, including North America (*n* = 2, 6.5%), South America (*n* = 1, 3.2%), Asia (*n* = 3, 9.6%), Africa (*n* = 2, 6.5%), and New Zealand and Australia (*n* = 2, 6.5%), and multi-continental research (Europe, Africa, and South America) (*n* = 1, 3.2%). Numerous factors can affect the productivity of continents in mental health research, and it is crucial to remember that research productivity is a complicated and multidimensional phenomenon. Some factors that could be responsible for regional differences in the productivity of mental health research include research infrastructure [18]; investment in research; cultural attitudes toward mental health [19]; education and training opportunities [20]; data accessibility, especially pertinent population-based data; policy and regulatory environment; international collaborations and funding [21]; and the time frame of the review, which might show significant difference in continental distribution if we altered the time window of the search. According to the study results, Europe took the lead in producing research relevant to psychiatric inpatient satisfaction, which indicates the availability of many of the above-mentioned elements in European countries and the increased concern about the quality of mental health services in this region of the world.

**Overview of the included studies:**

The samples of these studies mainly included psychiatric inpatients with various mental health conditions. The sample size of each study ranged from (*n* = 15) [22] to (*n* = 7302) [23]. More than half of the eligible studies (*n* = 20, 64.5%) were published in the last five years, and almost half of them (*n* = 14, 45.2%) used a cross-sectional study design. The remaining studies used various study designs such as pragmatic randomized trial (one study), exploratory study (three studies), quasi-experimental study (two studies), multi-center observational study (two studies), short semi-structured interviews (one study), two separate naturalistic trials (one study), qualitative study using a grounded theory (GT) design (one study), longitudinal, mixed-methods research project, using patients interviews with thematic analysis (one study), and pre–post study design (one study).

**Diagnosis:**

Most of the eligible studies in this scoping review included patients with common psychiatric disorders such as psychosis [22,24,25,26,27,28,29], affective disorder [24,30,31], anxiety disorder [22,29,31,32,33,34,35,36,37,38], bipolar disorder [26,36,39], depression [23,34,36,37,39,40,41,42], and drug-related disorders [29,33,34,42,43]. Some studies included personality disorders and eating disorders [26,34,44].

**Survey instruments for satisfaction assessment:**

Patient satisfaction questionnaires and different assessment scales were used in each study, including qualitative and quantitative questionnaires to assess inpatient satisfaction. Some studies [22,33,35,45,46] used the Client Assessment of Treatment (CAT) scale. Patient satisfaction is the main outcome of interest in the CAT scale; the scale is a seven-item survey that asks participants to assess how satisfied they are with various aspects of their inpatient treatment. In another study carried out in Norway [34], they described the creation and validation of the Psychiatric Inpatient Patient Experience Questionnaire—On-Site (PIPEQ-OS). The core of the PIPEQ-OS consists of three patient-assessed measures: structure and facilities (six items), patient-centred interaction (six items), and outcome (five items). In another study carried out in Norway [47], the UKU Consumer Satisfaction Rating Scale (the UKU-ConSat) was used to measure patient satisfaction at discharge and follow-up. The UKU-ConSat is provided as an interview with eight questions that investigate the patient’s experience with various elements of treatment and care. Mason satisfaction survey was created mainly as a quality improvement tool based on patient satisfaction with treatments [48]. The survey tool contained a Likert scale for each question ranging from ‘very important to me’ to ‘not at all’ to determine the relative significance of each topic to participants, as well as a final question bank of 50 questions organized into 14 separate subheadings. In some other studies [30,49], which focused on psychiatric patients’ satisfaction with different ward settings and door policies, all involuntarily committed patients with maintained mental ability were requested to complete the Zurich Satisfaction Questionnaire (ZUF-8), a German variant of the Client Satisfaction Questionnaire (CSQ). The Essen Climate Evaluation Scale (Essen-CES) was used to evaluate the ward atmosphere among other patients [25,30,50]. An exploratory cross-sectional study in Brazil employed the Brazilian Mental Health Services Family Satisfaction Scale (SATIS-BR). The World Health Organization created the SATIS-BR measure to evaluate satisfaction with mental health treatment in three groups: patients, relatives, and professionals. The measure consists of 13 items with five-point Likert scale responses. Greater scores indicate a greater level of satisfaction [26,51]. In another research study carried out in China, there was no worldwide patient satisfaction scale accessible in the Chinese language for mental patients; thus, the authors created a psychiatric inpatient satisfaction questionnaire for this study. The writers created the questionnaire based on a survey of the literature and expert comments. The psychiatric inpatient satisfaction surveys have traditionally included five domains: quality of treatment, interpersonal interactions, costs of care, non-medical services, and overall satisfaction. The Menninger Quality of Care (MQOC) measure was developed in collaboration with hospital treatment program directors in a study conducted in Texas, USA. The MQOC was designed to be concise, straightforward, useful, relevant, acceptable, and readily accessible. The authors offered descriptive and psychometric assessments of the measure, as well as a technique for using this information to support quality improvement activities [52]. Another multicentre trial that was carried out across eleven countries used a five-item study-specific questionnaire that was created in consultation with the leaders of each study site to gauge participant satisfaction. Q1. Did you think your hospital stay helped? Q2: How satisfied were you with the staff? Q3. Do you think anything happened to you while you were in the hospital? Q4: Were your rights and preferences taken into account? Q5: Was your privacy right upheld? All questions were translated into the participating nations’ native tongues by the site leaders [41].

**Factors associated with inpatients satisfaction with mental health services.**

**Ward atmosphere**

Efkemann et al. [30], Jovanović N et al. [35], Urbanoski et al. [38], and Chevalier et al. [32] assessed inpatient satisfaction against ward atmosphere and door policies, and they concluded that mixed-sex wards, ward renovation, and ward redesign to make family rooms off wards can potentially improve inpatients satisfaction; additionally, door control policies can impact voluntarily admitted patients’ satisfaction, but it has no significant effect on the satisfaction of involuntarily admitted patients.

**Mental illness severity and patient satisfaction**

Gebhardt et al. [25,44] and Kohler et al. [40] studied the effect of the severity of mental illness on patient satisfaction and found that patient satisfaction is mostly correlated with low severity of the mental disorder and a high level of global functioning at discharge.

**Staff–patient relationship**

MacInnes et al. [53], Jiang et al. [27], Zendjidjian et al. [54], Molin et al. [36,37] and Stewart et al. [55] studied staff–patient relationships and focused on the influence of this important factor on patient satisfaction, and they established the value of a good therapeutic connection between clinicians and service users, concluding that patients in the studied psychiatric inpatient care units were overall satisfied with their interaction with healthcare staff, although younger patients reported lower levels of satisfaction.

**Voluntary versus involuntary admission**

Cannon et al. [48], Soininen et al. [43], Zahid et al. [56], Bjertnaes et al. [34], Smith et al. [29], Bo et al. [24], and Ritsner et al. [57] evaluated patient satisfaction in the situation of voluntary versus involuntary admission including coercive measures such as seclusion and mechanical restraint. It was found that many patients felt that seclusion/restraint (S/R) was hardly necessary at all. Less satisfaction was reported by service users who were physically coerced, admitted involuntarily, and received less procedural justice. Higher levels of treatment satisfaction were linked to better functioning, enhanced insight, and therapeutic relationships. Older patients seemed to be against S/R. The results of this study suggested that coerced admission and incorrect or offensive treatment significantly affect satisfaction.

**Gender**

According to Bird V et al. [22] and Faerden et al. [58], men and women do not significantly differ in terms of service satisfaction or length of hospital stay; however, patients with personality disorders and short hospital stays are not as satisfied. During their study, Ratner et al. [59] found that when it came to “staff”, “care”, and general satisfaction, women were far less satisfied than men. Although most of the participants expressed satisfaction with the inpatient services, they felt that the areas of personal experience, knowledge, and activity were the weakest aspects of the program. Additionally, the authors claimed that five indicators correlated with satisfaction with hospital medical care: insight, physical health satisfaction, self-efficacy, family support, and social anhedonia.

**Cats in the ward**

Templin et al. [42] reported that patients living in wards with a cat had much greater overall satisfaction than patients living in wards without a cat; according to the authors, patients who lived in the company of a cat were also happier with the result of their therapy. Furthermore, they gave much higher ratings to their recreational options, common spaces, and teamwork with their main nurse, social worker, other therapists, and psychologists.

**Migration background and procedural fairness**

Gaigl et al. [39] discovered that patients with a migration history were more satisfied with their mental health care treatment than those without. Simultaneously, no variations in treatment utilization or real obtained mental healthcare were found between individuals with and without a migratory past. Regardless of treatment efficacy, Silva et al. [28] found that patients were happier with therapy if they felt it was delivered honestly and fairly. This finding, together with the discovery of a strong link between satisfaction with care and long-term treatment results, emphasizes the critical need to create treatments that increase the procedural fairness of psychiatric care.

**Forensic mental illness**

Forensic mentally ill patients are those who have interacted with the criminal justice system and have been sent to secure healthcare facilities. MacInnes et al. [53] sought to investigate how service users in a forensic mental health context perceive the therapeutic relationship with staff, how they perceive service satisfaction, and whether therapeutic relationship variables are connected with service user satisfaction in secure mental health facilities. The findings of this research highlighted the importance of developing a healthy therapeutic relationship between physicians and service users when measuring their satisfaction with the care and treatment they received in secure hospitals.

**Specific treatment components**

In order to investigate the satisfaction of individuals receiving treatment in mental health inpatient facilities, as well as to evaluate the viability of such services in multi-country clinical settings (eleven countries), Krupchanka et al. [41] carried out a cross-sectional international multi-centre study. The study’s findings showed that a large percentage of respondents were pleased with the inpatient care they received. Every study site had a positive skew in the satisfaction ratings. Paul et al. [23] and Stanton et al. [60] studied the comments of patients on a completed course of music therapy and physical activity for an MDD or an acute phase of SSD in a cross-sectional worldwide multi-centre research. The benefits of incorporating music therapy and physical exercise were documented as having fresh views, increased emotional fulfilment, being socially closer and more adept, and becoming free and artistically inspired, all of which favourably impacted overall patient satisfaction. Relevant and detailed information was extracted and summarized from the various studies and is presented in Table 1 below.

## 4. Discussion

This review has addressed patient satisfaction within inpatient mental healthcare settings. The review identified 31 eligible studies that fulfilled the inclusion and exclusion criteria. The results in the reviewed studies generally demonstrated high levels of respondents’ satisfaction with the inpatient services provided. The satisfaction scores were positively reported across many study sites. More than half of the eligible studies in this review were published in the last five years, which indicates a growing interest in measuring inpatient satisfaction as an important indicator for the quality of hospital services provided to psychiatric inpatients. The results suggest that inpatient satisfaction is mostly correlated with a low severity of the mental disorder, a high level of global functioning at discharge, and an improvement of the disease severity during the course of the treatment, and most patients tend to positively value staff members who maintain a good relationship and spend more time with them [22,27,44,48,53,61]. Similar results of generally satisfied patients were obtained in comparative studies carried out in different parts of the world, e.g., India [62], Kuwait [56], Kenya [63], Nigeria [64], Poland [65], Thailand [66], Finland [67], and Israel [68]. Additionally, unnecessary seclusion/restraint (S/R) of freedom was a significant source of dissatisfaction, and many patients felt that S/R was hardly necessary at all. Yet, they reported some benefits of S/R, such as helping them calm down and control agitation. Older patients seemed to be against S/R [33,38,46]. Other comparative literature also revealed the lack of involvement of the patient or a family member in the care plan or decision making was a prime source of reported patient dissatisfaction, and thus, a therapeutic alliance was a key factor in achieving optimal outcomes through addressing patients’ needs and providing the information that meets their needs. Patients with severe mental conditions or severe incompetency seemed less satisfied with hospital services, which, according to studies, could be attributed to their misjudgment and mental disability [59,69].

When placed in a hospital ward or other healthcare environment, cats and other animals can benefit the mental health of the patients and increase their satisfaction. Pet therapy or animal-assisted therapy are common terms used to describe this kind of treatment. Some studies provided explanations for why ward cats can help inpatients’ mental health, such as stress reduction, distraction from illness, reduction in feelings of isolation, emotional support, physical activity, and routine altering [42,70]. It is crucial to remember that although many people can benefit from animal-assisted therapy, it might not be appropriate for everyone. Healthcare settings must take into account various factors, including individual preferences, cultural beliefs, and allergies when implementing such programs. To safeguard the health of both patients and animals, appropriate cleanliness and infection control procedures should also be implemented [42,70,71,72]. Migration history and procedural fairness’s impact on mental inpatient satisfaction is a complicated and multidimensional subject that includes a range of elements linked to treatment outcomes, cultural diversity, and the general equity of the healthcare system. The way that procedural fairness and migration background interact is a key factor in determining how satisfied mental inpatients are. Important components include effective communication, cultural competency, and a procedure for treating people fairly and with respect [39,73]. Regardless of the origin of migration, mental health care providers and systems should work to establish an inclusive and culturally sensitive environment to increase overall patient satisfaction. Furthermore, continuing studies and satisfaction surveys conducted among a variety of patient populations can offer insightful information for future advancements in the provision of mental health services [39,73,74,75]. One of the most important ways to guarantee the standard of care and general well-being of patients in forensic mental health settings is to evaluate how satisfied forensic mental inpatients are with mental health services. Assessment and treatment of people with mental health disorders who are involved in the legal system are common components of forensic mental health services. Some ward procedures were found to be of great value in improving satisfaction in forensic psychiatric inpatients, for example, regular staff training, which ensures that healthcare personnel receive training in handling difficult behaviours, empathy, and effective communication. Feedback from family and friends is also an effective tool; their observations can provide a more comprehensive picture of the patient’s experience and point out any gaps in care or communication. Addressing safety concerns is an additional factor because inpatients with forensic mental health issues may face particular safety risks, and for their general satisfaction with the services as well as their well-being, it is imperative to create a safe and encouraging environment as well as provide patients with information about their medications, treatment regimens, and the objectives of their forensic mental health services in a clear and understandable manner. Patients who are well-informed and educated are more likely to feel engaged in their treatment [76,77,78,79].

Interestingly, when the studies are assessing the quantitative outcomes of inpatient satisfaction and the emphasis of the research studies is shifted from overall satisfaction to particular difficulties and experiences that psychiatric inpatients encounter, the overall picture drastically changes. For example, patients reported that physical/psychological abuse, staff misbehaviour [28,55,80,81], inadequate living conditions, and limited information availability were major sources of discontent [38,43,69,82]. The gap between overall satisfaction and many particular or specific issues during hospital stays should be addressed. When studies adopt a qualitative research methodology, the evidence of patient suffering is more visible. Open-ended surveys, focus groups, and interviews are a few examples of qualitative research methodologies that are used to investigate and comprehend the richness and depth of human experiences. These approaches enable a more thorough and nuanced analysis of patients’ viewpoints when used to measure patient satisfaction in healthcare, including mental health services. Qualitative research methods increase the visibility of patient suffering for a number of reasons, including rich narrative data, which are directly gathered from patients using qualitative methods. Patients can use open-ended questions to verbally describe their experiences, feelings, and perceptions. This narrative method offers a thorough and contextualized comprehension of the elements impacting their level of satisfaction or discontent. Another reason is that qualitative research can reveal aspects of care that may go unnoticed in quantitative studies by involving patients in candid discussions. Patients may disclose concerns, unfulfilled needs, or areas in need of improvement. This can involve problems with emotional support, communication, and general care quality [48,83,84,85,86,87,88]. In another context, expectations that were met or disregarded during the hospital stay play a crucial role in determining patient satisfaction because it is hypothesized that when expectations are met, people will be satisfied regardless of the calibre of care. Due to either the excellent care provided to the patient during their hospital stay or the mental inpatient’s low expectations and satisfaction threshold, for instance, when there is no chance of reaching their goal, a person may adjust to their surroundings by lowering their expectations [41,89]. Although the standards for each person in these facilities may differ, these environments usually have certain features in common, such as safety and security, respect for others, personal hygiene, medication management, family involvement, and discharge planning. Remember that exact expectations can change depending on the policies of the facility and laws in the area. Inpatient satisfaction has also been observed to be greater among elderly patients >44 years old. According to studies, older patients may be more adaptable to rigid ward routines, more compliant with treatment regimens, and more respectful, whereas younger patients may be more defiant, less accepting of their situation, and more resistant to staff instructions due to minor age differences [90,91]. Satisfaction with inpatient care has also been observed as lower among non-white patient groups in studies with a majority white population, which was ascribed in the research to the uneven level of services supplied to the white population [76,90,92]. Furthermore, in some additional studies, it was discovered that patients with poor mental health had lower short-term and long-term satisfaction than the rest of the patients; in this case, the studies assumed that impaired mental function and poor judgement were the main reasons for this observation [93,94]. Overall, there is evidence that mental inpatients are less happy than individuals released from the hospital after treatment of acute physical diseases [95]. There are several factors that may contribute to the perception that mental inpatients are less happy on average compared to other patients with physical diseases; for example, there is a strong social stigma associated with mental health issues, which can affect how people see themselves and are seen by others. Mental health patients may experience feelings of shame, loneliness, and low self-esteem as a result of this stigma. Another factor is lack of knowledge; as compared to physical illnesses, society may not be as sympathetic or understanding of mental health problems. This ignorance can make mental health patients feel more alone and make it more difficult for them to get the support they need. Furthermore, managing mental health conditions can present ongoing challenges as well as a risk of relapse. Mental health patients can experience anxiety and decreased levels of happiness as a result of this uncertainty and the possibility of setbacks. Additionally, treatment duration compared to the treatment of certain acute physical diseases, especially in an inpatient setting, may take longer. Prolonged absences from familiar surroundings and one’s home can exacerbate feelings of unease and discontent [96,97,98,99,100]. Patient satisfaction is a wide statistic that is often used to evaluate general healthcare interventions in the context of mental health treatment [30]. Despite a large body of research on inpatient satisfaction in mental health services, there are still some noteworthy research gaps that call for more study. By identifying and filling these gaps, we can improve the delivery of mental health care by gaining a more comprehensive understanding of the factors influencing patient satisfaction. The following are some possible research gaps in the assessment of inpatient satisfaction. Diversity and cultural sensitivity: The effects of diversity and cultural factors on inpatient satisfaction have received little attention in the literature. Developing culturally sensitive care models requires examining how cultural differences affect patient expectations, preferences, and satisfaction with mental health services [101]. Patients’ expectations, prior treatment experiences, and knowledge about services should all be taken into consideration in future research on patient satisfaction. Patient-reported outcome: Results as reported by patients are in need of more research that directly incorporates patient-reported outcomes, as many studies currently rely on provider- or system-reported outcomes. For a thorough assessment, it is imperative to evaluate satisfaction from the patient’s point of view, taking into account their opinions about communication, participation in decision making, and overall experience [102]. Integration of mental and physical health services: Health Studies frequently concentrate on mental health services separately, but the significance of combining mental and physical healthcare is becoming increasingly acknowledged. There is growing interest in learning how the integration of these services affects overall health outcomes and inpatient satisfaction. Peer support’s impact: Further research is needed to fully understand the impact of peer support on inpatient satisfaction. Examining the impact of peer support programs on patient experiences, satisfaction, and engagement in inpatient mental health settings can yield important information for enhancing care [103,104,105]. Telehealth and technology: Research on the effects of telehealth and technology on inpatient satisfaction is necessary, as these modalities are increasingly being used in mental health services. It is necessary to investigate how virtual interactions affect patient perceptions and satisfaction in comparison to traditional in-person care [106,107]. And finally, instruments and procedures for measurement: Measurement instruments that are validated and standardized are required for evaluating inpatient satisfaction in mental health settings. Furthermore, investigating cutting-edge research techniques like mixed methods and qualitative approaches can offer a more complex understanding of patient experiences [101,102,103,106]. The goal of this scoping review was to explore patient satisfaction in mental health inpatient facilities by explicitly evaluating such services from a patient viewpoint and analyzing their practicability in multi-country clinical settings. Using this technique for service evaluation, we were able to efficiently produce patient responses from all over the world and collect data for the aforementioned purpose. We were also able to identify the gaps in the current research works that need to be further addressed in future research. The majority of the studies used in this scoping review adopted a pre-existing inpatient satisfaction questionnaire or developed a new one, which seemed to be effective and acceptable in quality. It would also be useful for future studies to explore patient satisfaction with care by using other technological measures like electronic surveys, which seem to be easier and less time-consuming for patients to complete, as well as for the research team to interpret the results by using data analysis software like SPSS version 29 [107,108]. Currently, a pragmatic stepped-wedge cluster-randomized trial is running in the province of Alberta, Canada, where the researchers meet with patients who are about to be discharged from the psychiatric units in the main hospitals all over the province and they invite them to fill out a satisfaction survey digitally, using an online link that includes questions related to their hospital experience and the degree of satisfaction with the services provided to them. In this multi-centre trial, the research team provides two main interventions: daily supportive text messages (text for support) and mental health peer support [109]. It would be useful to expand the focus of future studies and reviews to explore the satisfaction of psychiatric patients with novel and productive services, such as peer support programs and other technological supportive measures, such as text for support, which has demonstrated high effectiveness among patients [107,108,110,111].

According to the research we reviewed, we strongly recommend that clinicians and other healthcare professionals consider the following points to achieve better therapeutic alliance and satisfaction of their inpatients: a clear discharge plan, less coercive treatment during the hospital stay, more personalized higher quality information and teaching conducted by staff to patients about the mental disorders, and specific treatment components that are well perceived by patients, such as physical exercise sessions and music therapy, which all appear to be associated with higher inpatient satisfaction with care. Ward atmosphere and design are also crucial considerations. Personal contact, such as improved therapeutic connections with staff, particularly nurses, is another crucial element.

## 5. Limitations

The authors of this scoping review are aware of various limitations. First, we exclusively examined English language databases for this scoping review. Considering our criteria, great care was taken to find all pertinent studies for this evaluation. We could, however, have overlooked some important research, particularly those that were written in different languages. Non-English language studies with negative or null findings may go unpublished. This could result in a skewed representation of the available evidence. This limitation could be mitigated in future research, like conducting a broader search that includes databases in other languages. Additionally, this review did not assess the risk of bias or provide a meta-analysis outcome; given that the different nature of study outcomes, including qualitative information, measurement scales, and questionnaires from diverse geographical regions, this might potentially cause variations in the evaluations and outcomes. We suggest that future research may consider a more in-depth analysis of the risk of bias or conduct a meta-analysis if feasible. Finally, aiming to cover the recently updated research evidence, this review covered a certain time frame for searches in the literature, which could have overlooked other valuable previous research work carried out before this period. We encourage future researchers to consider a more extensive time frame to ensure a comprehensive review of the literature.

## 6. Conclusions

This scoping review highlighted the generally high levels of patient satisfaction within inpatient mental healthcare settings. The findings indicate a growing interest in measuring inpatient satisfaction as a crucial indicator of the quality of hospital services provided to psychiatric inpatients. Patient satisfaction is notably correlated with factors such as the severity of the mental disorder, global functioning at discharge, improvement during treatment, and positive interactions with staff. This review underscored the positive impact of interventions such as animal-assisted therapy or pet therapy for mental health and patient satisfaction, but it also highlighted the need to implement these programmes with consideration for individual preferences and cultural sensitivity. Furthermore, the complex relationship between migration history and procedural fairness on mental inpatient satisfaction is explored, emphasizing the importance of effective communication, cultural competency, and fair treatment. This review notes that although overall satisfaction is high, it is important to address certain problems that psychiatric inpatients face, like abuse, staff misbehaviour, living conditions, and information availability. The use of qualitative research methods was highlighted for a nuanced understanding of patient experiences and satisfaction, revealing aspects that may be overlooked in quantitative studies. Several factors influencing satisfaction were identified in this review, including age, cultural background, mental health conditions, societal stigma, and treatment duration. This emphasizes the need for future research to fill gaps in understanding diversity, cultural sensitivity, patient-reported outcomes, integration of mental and physical health services, the impact of peer support, and the effects of telehealth and technology on inpatient satisfaction. In addition to offering insights into the current status of inpatient satisfaction in mental health facilities, the scoping review identified important areas for relevant ongoing research. The ongoing stepped-wedge cluster-randomized trial in Alberta, Canada, using digital surveys and interventions like daily supportive text messages and mental health peer support, exemplifies the evolving landscape of research methodologies to further explore and enhance psychiatric patient satisfaction.

## Figures and Tables

**Table 1:** Summary of studies examining inpatient satisfaction with mental health services.

Author (Year)	Type of Study	Country of Study	Diagnosis	Number of Participants	Aim of the Study	Results and Conclusions
Bird V. et al., 2020 [31]	Cross-sectional	Five European countries (Germany, UK, Italy, Poland, Belgium)	Psychosis, affective disorder, anxiety disorder	7302 patients	The purpose of this research is to address past constraints by determining whether patient characteristics are connected with satisfaction in a large sample of mental inpatients across five European nations.	This research tries to address prior constraints by determining whether patients being older, working, living with others, having a close friend, having a less serious sickness, and being admitted for the first time all contributed to higher satisfaction ratings. Higher levels of schooling, concomitant personality disorder, and involuntary admission, on the other hand, were related to lower levels of satisfaction. Although the same patient variables predicted satisfaction across the five nations, there were substantial disparities in total satisfaction levels. Patients in the United Kingdom were highly dissatisfied with their inpatient treatment when compared to other nations.
Bird V. et al., 2018 [22]	Cross-sectional	UK	Psychosis, affective disorder, anxiety disorder	Total of 2709 patients, 1612 functional and 1097 sectorial	Patient satisfaction and duration of stay, as well as functional and sectorized care.	In all, 2709 patients were enrolled, with 1612 receiving functional care and 1097 receiving sectorized care. Patient satisfaction in sectorized treatment was considerably greater (Mean difference = 0.54, 95% CI = 0.35–0.73, *p* < 0.001).
Bjertnaes et al., 2018 [34]	Cross-sectional	Norway	Anxiety, depression, drug-related disorder, eating disorder	1683 patients	The present research sought to investigate the significance of several forms of patient-reported predictors for mental health inpatients’ evaluations of outcomes, including both background characteristics at the patient level and healthcare predictors linked to healthcare structure and procedures.	A variety of organizational and healthcare characteristics were linked to patient-assessed outcomes, the most significant of which were clinicians/staff understanding your position, therapy tailored to your circumstances, and enough information about your mental health condition. Coerced admission and inappropriate or insulting treatment have a considerable negative influence on satisfaction.
Bo 2016 [24]	Pragmatic randomized trial	Norway	Active psychosis	226 patients	The goal was to measure satisfaction among acutely hospitalized patients with psychosis, to compare satisfaction in voluntarily vs. involuntarily admitted patients, and to examine the impact of symptom burden and insight.	The great majority of acutely hospitalized patients were pleased with their care, except for the fact that the involuntary care group was obviously less happy with the information supplied. Generally, there were little differences between the involuntary and voluntarily admitted patient groups. In psychosis, poor insight has a significant detrimental influence on treatment satisfaction. The supply of appropriate and acceptable information is a key goal for improving mental health care services.
Cannon 2018 [48]	Quantitative and qualitative cross-sectional	New Zealand		541 out of 1034	The primary goal is to assess patient satisfaction after the acute episode of psychosis. The secondary goal is to compare satisfaction levels in voluntary and involuntary admissions.	The vast majority of people were happy with hospital care; however, the forced admission group was definitely dissatisfied.
Chevalier 2018 [32]	Short semi-structured interviews	UK	Psychosis, mood disorder, anxiety disorder	61 patients, 20 female and 41 male	The goal was to investigate the first hospitalization experience. A secondary goal of the research was to investigate what could underpin both very favourable and extremely negative perceptions of hospital treatment and care.	Reducing the effect of uncertainty and fostering positive connections may assist services in improving the first experience of hospital admission and, eventually, improving patient outcomes.
Efkemann 2019 [30]	Part of a larger mixed methods study consisting of a quantitative and qualitative subproject	Germany	Substance disorders, psychotic disorders, affective disorders	Hospital 1 (locked): 632 patients; Hospital 2 (facultative locked): 106 patients; Hospital 3 (open): 28 patients	The study’s goal was to learn more about the connections between various door policies, ward environments, and patient happiness.	In the unique group of patients under involuntary commitment, important characteristics of the ward environment seem to be better in an open vs. a locked setting, although patient satisfaction does not appear to be impacted by the door status. However, door control rules cause anxiety and unhappiness among voluntarily committed patients.
Faerden 2020 [58]	Survey study	Norway	Every patient admitted to the Department of Acute Psychiatry, with the exception of those who were diagnosed as mentally retarded and those who were unable to communicate in Norwegian	256 patients	Seeks to assess potential differences in patient satisfaction between sexes, days spent in the hospital, diagnostic groups, patients admitted voluntarily and involuntarily based on hospital data, and patients’ perceptions of voluntary and involuntary admissions.	There was no statistically significant difference in PS between men and women; however, patients with personality disorders and brief stays were less happy. Regardless of legal status, PS was much lower for individuals who perceived involuntary admission.
Gaigl 2022 [39]	Cross-sectional study	Germany	Schizophrenia and depression Bipolar disorder	398 patients participated in the study	Multicentre research was conducted to examine the amount and quality of therapy among patients with and without a migration background.	According to the research, individuals with a migration history were more satisfied with their mental health care treatment in the previous 12 months than those without. Simultaneously, no variations in treatment utilization or the degree of agreement between required and obtained mental healthcare were found between individuals with and without migrant origin. Patient satisfaction findings are consistent with those of Canadian research, which found that first-generation migrants are more satisfied with mental health treatment than native Canadians.
Gebhardt 2013 [44]	Cross-sectional	Germany	-Organic disorders - Schizophrenia - Schizotypal and delusional disorders - Mood affective disorders - Neurotic and stress-related disorders - Somatoform disorders - Behavioural syndromes associated with physiologic disturbances and physical factors disorders of adult personality Mental retardation	113 patients: 42 patients were admitted by their general practitioner, 13 by their psychiatrist, 13 by the clinic’s outpatient department, 6 by an emergency physician, 23 patients requested admission by themselves, 8 were transferred from another ward, 3 were transferred from another clinic, and 5 were admitted for other reasons	The current research attempts to investigate connections between patient satisfaction and (1) treatment factors and treatment effects, (2) characteristics associated with fundamental disorders, and (3) sociodemographic factors.	According to the study’s findings, patient satisfaction is generally associated with a low severity of the mental condition, a good level of global functioning after discharge, and a decrease in disease severity throughout treatment. Furthermore, pharmacologic disruptions and association with a schizophrenia spectrum or personality disorder seem to reduce patient satisfaction.
Godoy 2019 [26]	Exploratory cross-sectional study	Brazil	Bipolar disorder; Unipolar depression; Personality disorders; Psychotic disorders; Substance use/misuse; Neurotic and anxiety disorders	257 patients	The purpose of this study is to assess patient satisfaction with treatment and care in a sample of inpatients with mental disorders treated at a general hospital, as well as to examine the relationships between patient satisfaction with clinical treatment and sociodemographic variables, specifically the type of health insurance (public vs. private).	The patients were pleased with their care in the mental inpatient facility. Satisfaction was mostly connected to patients’ perceptions of progress and was higher in the public health system group. Treatment satisfaction assessment is an essential result in health and should be integrated into the reorientation of changes in organizational procedures, team training, and physical design improvements.
Guzman-Parra 2019 [33]	Quasi-experimental study	Spain	Substance disorders, psychotic disorders, affective disorders, anxiety disorders, and personality disorders	111 patients were included in the final analysis	The purpose of this study was to examine patients’ perceptions of coercion, symptoms of post-traumatic stress disorder, and subjective satisfaction with hospitalization treatment associated with the use of various coercive measures during psychiatric hospitalization, particularly the use of involuntary medication, mechanical restraint, or a combination of these measures. The coercive measure was used at least 48 h prior to the evaluation.	There were considerable disparities in the three groups’ perceptions of coercion. With a stronger perception of compulsion in the group subjected to combination measures, the use of involuntary medicine was linked to decreased levels of perceived coercion and post-traumatic stress disorder. Furthermore, the combination of mechanical constraint and forcible medicine was linked to decreased levels of satisfaction with medical care. Following the use of involuntary medicine, mechanical restraint, or a combination of these methods, there was a substantial incidence of event-related post-traumatic stress disorder.
Jiang 2019 [27]	Cross-sectional survey	China	Schizophrenia and associated conditions (47.8%) Mood disturbances	The final analysis included 1663 patients. The participants’ average age was 41.9 years, with 51.7% of them being female, 52.6% being married, and 26.1% having completed college or higher education	This study looked into the clinical and institutional determinants of satisfaction as well as the level of satisfaction among psychiatric patients.	In conclusion, a nationwide study conducted on mental inpatients in China found that most of the respondents were happy with the care they received. Furthermore, we identified a number of potential factors influencing patient satisfaction in China. Higher satisfaction levels were correlated with individual characteristics like better education and treatment response, as well as with institutional characteristics like adequate staffing.
Jovanovic 2022 [35]	Quasi-experimental study	UK and Italy	The majority of patients were diagnosed with psychosis (*n* = 877, 41.2%) Affective disorders Anxiety disorder	The research comprised 2130 people, 1430 (67.1%) from England and 700 (32.9%) from Italy. The sample was gender balanced, with 1186 (55.8%) males and 940 (44.2%) women participating. Their average age was 41.8 years (standard deviation: 13.9)	The purpose of this research is to determine which aspects of the hospital environment are connected with improved patient satisfaction with mental inpatient treatment.	Because hospitals are among the most costly structures to construct, their design should be informed by research findings. Family rooms off wards and mixed-sex wards are two design aspects that may increase patient happiness.
Kohler 2015 [40]	Two separate naturalistic trials	Germany	Major depressive disorder Schizophrenia or related disorder	The research involved 356 participants. The study comprised 75 individuals with schizophrenia and 217 participants with unipolar depression	The purpose of this research is to analyze and compare patient satisfaction in an inpatient psychiatric environment between patients with MDD and patients with schizophrenia.	There were no variations in patient satisfaction between MDD and schizophrenia. The severity of the illness and comorbidities in MDD, as well as the quantity of medicines administered in both groups, were associated with worse patient satisfaction.
Krupchanka 2017 [41]	Cross-sectional international multi-centre survey	Total of eleven countries, including - seven European countries (Bosnia and Herzegovina, Czech Republic, Italy, Romania, Ukraine Russia, and Croatia), - three African countries (Tunisia, Uganda, and Nigeria), and - one South American country (Argentina)	Of the patients, 29% were diagnosed with depression and 35% with schizophrenia. And other/multiple disorders make up the remainder. 14% of them were lawfully detained at the time of admission	Following the removal of 28 (4.0%) respondents whose satisfaction questionnaire data were missing, 673 people made up the final research sample. Males made up 56% of the population, followed by married couples (38%), college graduates (37%), and working adults (29%)	The goal of the study is to examine how satisfied patients are with the services they receive in inpatient mental health facilities from a patient’s point of view and to examine how well these services are implemented in multi-national clinical settings.	The study’s findings revealed that respondents were quite satisfied with the inpatient care provided. Across all research sites, satisfaction levels were biased favourably. These findings are consistent with past research that found high levels of patient satisfaction with mental health care.
MacInnes 2014 [53]	Cross-sectional survey design	UK	Not specified	77 people took part. The bulk of responses were male (84%) with 12 (16%) females. In terms of ethnicity, the most often documented was White British, while twenty people identified as Black British, African, or Caribbean.	The study’s goal was to look into the following questions: How do service users perceive the therapeutic connection with personnel in a forensic mental health setting? How do service consumers perceive satisfaction with care in a forensic mental health setting? What aspects of therapy relationships are related to service user satisfaction in safe mental health settings?	The findings of this research highlight the importance of creating a healthy therapeutic relationship between doctors and service users when evaluating their satisfaction with the care and treatment they received in secure settings. Mentally ill offenders are individuals who have dealt with the criminal justice system and have been sent to secure institutions or transferred to secure hospitals.
Madan 2014 [52]	Exploratory study	Huston, Texas, USA	Not specified	337 adult inpatients took part in the study. In terms of demographics, the research sample was mostly composed of the following: - Young adults; - Race white (92.28%); - Gender female (51.93%); - Education 16 years or more (52.52%), but not working in the 30 days before the hospitalization (62.61%) - The rates of marital status were single (58.16%), never married (22.85%), married (22.85%) divorced (12.17%), separated (4.75%), widowed (1.78%), and cohabiting (0.3%)	The creation and preliminary psychometric features of the MQOC, as well as a technique for exploiting patient satisfaction data for targeted quality improvement activities, are discussed in this article.	Overall, 91.7% were somewhat or very happy with their treatment (QOC total score 60). The overall quality of care received by patients was scored as follows: 7/10 or higher (93.2%), 8/10 or higher (86.4%), 9/10 or higher (71.3%), and 10/10 (31.2%). Patients evaluated the facility’s probability of recommendation as 7/10 (89.0%), 8/10 or better (84.4%), 9/10 or better (74.2%), and 10/10 (49.0%).
Molin 2016 [36]	Qualitative study using a grounded theory (GT) design	Sweden	Diagnoses made by the patients themselves included dissociative syndrome, anxiety, burnout, eating disorders, bipolar disorder, depression, posttraumatic stress disorder, Tourette’s syndrome, and borderline personality disorder. Four people reported not knowing about their condition	A total of sixteen individuals, two men and fourteen women, from five distinct wards participated. Their ages ranged from 20 to 51 years old, with 31 serving as the median	The purpose of this research was to investigate daily life in mental inpatient treatment through the vision of patients.	Quality interactions, defined as closeness to staff in daily relationships and spending quality time on basic tasks, would enhance patients’ experiences of everyday life in mental inpatient treatment and, hence, help their recovery. The personnel must reconsider their priorities by thinking about the kind of activities they participate in. Paying attention to the little things may improve the quality of relationships and aid in the creation of a recovery-friendly atmosphere.
Molin 2021 [37]	Cross-sectional study	Sweden	The majority of participants met the criteria for probable or possible depression or anxiety	There were 84 participants in all (42 women, 38 men, and 4 missing data)	The purpose of this research was to assess patients’ satisfaction with their interactions with psychiatric inpatient care (PIC) personnel and to see whether sociodemographic characteristics, depression, and anxiety symptoms had a role in their perceptions of these interactions.	In general, patients in the assessed psychiatric inpatient care (PIC) units expressed satisfaction with their most recent interactions with medical staff; however, younger patients expressed less satisfaction.
Paul 2020 [23]	Exploratory study	Pretoria, South Africa	Eight MDD and seven acute phases of SSD	Six male and nine female subjects were purposefully sampled after completing a course of time-limited individual music therapy during their hospitalisation	The study’s goal was to investigate the benefits of music therapy as described and experienced by clients.	The research looked at patients’ perspectives on a completed course of music therapy for MDD or an acute episode of SSD. Gains were defined as having new views, becoming stronger, having emotional fulfilment, becoming socially closer and more adept, and being emancipated and artistically inspired.
Ratner 2018 [59]	Cross-sectional	Israel	Schizophrenia (81 patients) and schizoaffective disorder (44 patients) in a stable condition	The study comprised 125 consecutive inpatients with schizophrenia or schizoaffective disorder who were in stable condition. There were 96 males; 77 women; 11 married people; and 37 divorced, separated, or widowed people. At the time of the evaluation, none of the individuals’ mental or physical illnesses had worsened	This research investigated a broad range of topics concerning mental hospital service satisfaction among people with SZ/SA illnesses in order to identify markers connected with patient perception.	Personality traits, rather than psychopathological symptoms, were linked to patient satisfaction with treatment in patients hospitalized with severe mental illness. These variables might be the focus of efforts aimed at improving treatment and hospital services. Although participants were generally satisfied with the inpatient treatments, they said that the weakest components of the programme were in the realms of “personal experience”, “information”, and “activity”. Women were substantially more unsatisfied with ‘staff’, ‘care’, and overall satisfaction than males. - Satisfaction with hospital health treatment was linked to five indicators: insight, physical health satisfaction, self-efficacy, family support, and social anhedonia.
Ritsner 2018 [57]	Cross-sectional	Israel	Schizophrenia (81 patients) and schizoaffective disorder (44 patients) Patients were admitted to a hospital either by district psychiatrist order (DPO), court observation order (COO), or voluntary (VA).	The study comprised 125 consecutive inpatients with schizophrenia or schizoaffective disorder who were in stable condition. There were 96 males; 77 women; 11 married people; and 37 divorced, separated, or widowed people. At the time of the evaluation, none of the individuals’ mental or physical illnesses had worsened	The goal of this cross-sectional research was to examine three groups of hospitalized patients with serious mental problems who were admitted forcibly and those who were admitted willingly.	Among the 125 individuals included, 38 were VA admitted, 49 were DPO admitted, and 38 were COO admitted.
Silva 2022 [28]	Cross-sectional	Switzerland, French-speaking Swiss cantons	Disorders of adult personality and behaviour Disorders of psychological development Mental and behavioural disorders due to psychoactive substance use Schizophrenia, schizotypal, and delusional disorders Mood affective disorders Neurotic, stress-related and somatoform disorders	133 voluntary and involuntary admitted inpatients were interviewed	The research hypothesized that when treatment demands were viewed as fair and successful, patients were more happy with their care, even if they were subjected to formal coercion or had previously undergone formal coercion. The primary goal of this research was to put this idea to the test on a group of voluntary and involuntary mental inpatients.	According to this research, patients were more happy with therapy if they believed it was administered equitably, regardless of its efficacy. This finding, together with the discovery of a strong link between satisfaction with care and long-term treatment results, emphasizes the critical need to create treatments that increase the procedural fairness of psychiatric care.
Smith 2014 [29]	Multi-centre observational study	Ireland	- Anxiety and affective disorders - Psychotic disorders; co-morbid diagnoses - Alcohol—harmful use or dependence - Cannabis—harmful use or dependence	129 voluntary and involuntary admitted inpatients were interviewed, 69 males and 60 females	The purpose of this research was to assess service satisfaction in a representative inpatient sample after admission to a psychiatric hospital and to identify demographic, clinical, and service characteristics related to satisfaction.	Overall, service satisfaction was high (CSQ-8 mean score of 24.5). Service users who were admitted unwillingly, who were subjected to physical coercion, and who received lower levels of procedural fairness were less pleased. Higher levels of treatment satisfaction were connected with a stronger therapeutic connection, increased insight, and better functioning.
Soininen 2013 [43]	Cross-sectional study	Southern Finland	Alcohol abuse patients Schizophrenia patients Affective disorder patients Personality disorder patients	90 patients, 55 men and 35 women, all experienced S/R from 75 min to 16 days - Restraint 40 - Seclusion 26 - Both 24	The study’s goal was to describe the views of care of S/R patients throughout their hospital stay. Furthermore, the researchers intended to find out what elements were linked to patients’ evaluations of their treatment.	In the current research, patients thought that S/R was not at all essential. Nonetheless, they reported some S/R advantages. Older individuals, in particular, seemed to be opposed to S/R. According to the findings of this research, patients’ perspectives were not included in treatment planning. This might indicate that paternalistic decision-making persists in mental institutions or that there is a lack of open and respectful communication between patients and healthcare workers. It is possible to infer that mental therapy should be created on a more ethical foundation. We must enable patients to express their concerns and participate in their care.
Stanton 2016 [60]	Cross-sectional study	Australia	All inpatient mental health patients released between January and March 2014 were deemed eligible for participation in this study	Total of 32 inpatients completed discharge surveys to evaluate group activities	Attendance and satisfaction with a group fitness programme in an inpatient mental health environment are investigated in this research.	When compared to all other activities, exercise was evaluated as “excellent” by more inpatients (*n* = 16, 50%). Nonattendance rates for cognitive behavioural treatment were lowest (*n* = 2, 6.3%), highest for the relaxation group (*n* = 6, 18.8%), and 12.5% (*n* = 4) for the group exercise programme.
Stewart 2015 [55]	Longitudinal, mixed-methods research project using patient interviews with thematic analysis	UK	No specific psychiatric diagnosis was reported in eligible patients	Total of 119 patients were interviewed	The purpose of this study was to explore patient impressions of nursing staff by taking into account both personal and professional traits of nurses as well as their contribution to the ward environment and to apply this to a far bigger sample than most prior research has managed.	The results provide the sense of uneven and often poorly conveyed treatment, which cannot be considered good, but they also indicate where efforts to enhance the patient experience should be directed. There may be potential to increase professional nursing skills training and growth, but this ignores the fundamental social skills that many patients in the sample thought were missing.
Templin 2018 [42]	Retrospective study	Switzerland	Psychosis, depression, or substance abuse	170 inpatients, 46.50% were women	The research looked at the association between the presence of cats in psychiatric wards and satisfaction of inpatients suffering from depression and drug addiction in stationary psychiatric treatment.	Those in wards with cats reported much better overall satisfaction than those in wards without cats. Patients who lived in the company of a cat were also happier with the result of their therapy. Furthermore, they evaluated their recreational options, common spaces, and cooperation with their main nurse, social worker, other therapists, and psychologists as being substantially higher, but the collaboration with the doctor had no influence. Patient satisfaction with their accommodations, meals, and cafeteria did not change across wards with and without cats.
Urbanoski 2013 [38]	Pre–post study design	Toronto, Canada	Mood and anxiety disorders	290 adults	The purpose of this research was to see whether ward environment mediated the relationships between unit redesign and patient outcomes, such as treatment satisfaction and improvements in mental health-related quality of life and functioning throughout treatment. The authors hypothesized that following the makeover, patients would report a more pleasant ward environment, which would result in improved outcomes.	Participants recruited after the redesign performed better at admission than those recruited before the makeover. There were no gender, age, work position, diagnosis, or quality of life disparities. Understanding how architectural elements and clinical processes impact the psychosocial environment of inpatient units and patient outcomes is crucial for good therapeutic space design.
Zendjidjian 2014 [54]	Cross-sectional study	Marseille, France	Schizophrenia 91 (34.3%) Mood disorders 140 (52.8%) Other 34 (12.8)	265 patients agreed to participate in the study. 138 were men	Using a specialized, self-administered questionnaire, the goal of this research was to identify patient and care-related characteristics that are connected with patient satisfaction with mental hospital treatment.	For the first time, a specialized, multidimensional satisfaction questionnaire based only on patients’ points of view was used in this research to identify many possible factors of satisfaction. The most critical factors related with patient satisfaction were the therapeutic interaction and isolation.

## Data Availability

Data supporting the conclusions of this article will be made available by the authors without undue reservation.
